# Do we need to demonstrate Amyloid in tissue?

**DOI:** 10.1186/1750-1172-10-S1-I13

**Published:** 2015-11-02

**Authors:** Laurent Magy

**Affiliations:** 1 Service et Laboratoire de Neurologie, Centre de Référence "Neuropathies Périphériques Rares", CHU Limoges, France

## Background

Famililal amyloid polyneuropathy due to mutations in the Transthyretin gene (TTR FAP) is a constantly disabling condition leading to death from cardiac or renal failure, or cachexia linked to end-stage neuropathy with autonomic involvement. It has an autosomal dominant inheritance and according to the literature, the phenotypic presentation has a bimodal age of onset with early onset cases occurring before 50 years of age and so-called late onset cases occurring thereafter. To date, therapeutic options are proposed to people who carry a TTR mutation and have evidence of organ involvement. Additional histopathologic demonstration of amyloid deposits in a tissue sample is often considered mandatory to start treatment, although this relies more on professional agreement than on medical or scientific evidence.

## Methods

We reviewed the literature (PubMed Search and personal files) on TTR FAP with an emphasis on the requirements for amyloid deposit demonstration for its diagnosis in individuals with or without previous evidence of a mutation in the TTR gene. Additionally, we looked for available evidence for amyloid deposit demonstration in TTR mutation carriers before initiating treatment. Finally, we reviewed the diagnostic tools that are available to detect any meaningful change in the peripheral (autonomic and somatic) nervous system in patients at risk for peripheral neuropathy, especially in TTR mutation carriers.

## Results

The diagnosis of TTR FAP in patients with an apparently sporadic peripheral neuropathy is said to require the presence of a mutation in the TTR gene AND evidence of amyloid deposit in at least one of the following tissues: labial salivary gland, abdominal fat aspirate, gastro-intestinal tract and (sural) nerve. However, we found surprisingly low scientific evidence to support the mandatory demonstration of amyloid in any tissue in individual with peripheral neuropathy AND TTR mutation after excluding any other possible cause of neuropathy. This issue is very important in the genetic era because we are increasingly aware of "asymptomatic" TTR mutation carriers and there is still no consensus on the adequate follow-up for these patients (in terms of timing and investigations tools).

We found a wide variety of available tools to detect somatic and autonomic peripheral neuropathy in TTR mutation carriers. Based on the available literature, some proposals may be done in order to diagnose as early as possible peripheral neuropathy in these individuals.

## Conclusion

Available literature shows that the need for demonstration of amyloid deposit in any tissue in patients with peripheral neuropathy and a mutation in the TTR gene is more a dogma than an evidence-based requirement. Although search for amyloid deposits should be conducted when amyloid neuropathy is suspected, we think people who carry a TTR FAP mutation and have reasonable evidence of peripheral neuropathy should not be denied access to effective therapy, even if no demonstration of amyloid deposit can be done.

